# Long-Term Results after Proximal Thoracic Aortic Redo Surgery

**DOI:** 10.1371/journal.pone.0057713

**Published:** 2013-03-01

**Authors:** Martin Czerny, Ilan Barchichat, Katharina Meszaros, Gottfried H. Sodeck, Alberto Weber, David Reineke, Lars Englberger, Florian Schönhoff, Alexander Kadner, Hansjörg Jenni, Jürg Schmidli, Thierry P. Carrel

**Affiliations:** 1 Department of Cardiovascular Surgery, University Hospital Berne, Berne, Switzerland; 2 Department of Cardiac Surgery, Medical University Graz, Graz, Austria; 3 Department of Emergency Medicine, Medical University of Vienna, Vienna, Austria; Sapienza University of Rome, Italy

## Abstract

**Objective:**

To evaluate early and mid-term results in patients undergoing proximal thoracic aortic redo surgery.

**Methods:**

We analyzed 60 patients (median age 60 years, median logistic EuroSCORE 40) who underwent proximal thoracic aortic redo surgery between January 2005 and April 2012. Outcome and risk factors were analyzed.

**Results:**

In hospital mortality was 13%, perioperative neurologic injury was 7%. Fifty percent of patients underwent redo surgery in an urgent or emergency setting. In 65%, partial or total arch replacement with or without conventional or frozen elephant trunk extension was performed. The preoperative logistic EuroSCORE I confirmed to be a reliable predictor of adverse outcome- (**ROC 0.786, 95%CI 0.64–0.93**) as did the new EuroSCORE II model: **ROC 0.882 95%CI 0.78–0.98**. Extensive individual logistic EuroSCORE I levels more than 67 showed an **OR** of **7.01**, **95%CI 1.43–34.27**. A EuroSCORE II larger than 28 showed an **OR** of **4.44** (**95%CI 1.4–14.06**). Multivariate logistic regression analysis identified a critical preoperative state (**OR 7.96, 95%CI 1.51–38.79**) but not advanced age (**OR 2.46, 95%CI 0.48–12.66**) as the strongest independent predictor of in-hospital mortality. Median follow-up was 23 months (1–52 months). One year and five year actuarial survival rates were 83% and 69% respectively. Freedom from reoperation during follow-up was 100%.

**Conclusions:**

Despite a substantial early attrition rate in patients presenting with a critical preoperative state, proximal thoracic aortic redo surgery provides excellent early and mid-term results. Higher EuroSCORE I and II levels and a critical preoperative state but not advanced age are independent predictors of in-hospital mortality. As a consequence, age alone should no longer be regarded as a contraindication for surgical treatment in this particular group of patients.

## Introduction

Persisting or recurring aortic pathology in a proximal thoracic aortic segment after previous repair of acute or chronic aortic pathology (dissection or aneurysm) is increasingly observed. According to the individual pathological process- progression of the disease within the aortic root, progression of disease in the aortic arch, infection, pseudoaneurysm or a combination of these processes, strategies for effective treatment must be clearly defined [1]–[4]. Literature available reports on heterogenous patient cohorts with variable primary operations. This makes an objective evaluation of current results of these operations necessary [5].

The aim of this study was to evaluate our institutional results in patients who underwent proximal thoracic aortic redo surgery.

## Patients and Methods

### Patients

We analyzed 60 patients (median age 60 years, median logistic EuroSCORE 40) who underwent proximal thoracic aortic redo surgery between January 2005 and April 2012. Early and mid-term outcome as well as risk factors for mortality were analyzed. Inclusion criteria for this study were any type of previous proximal thoracic aortic surgery including root, ascending or aortic arch repair. Patients undergoing proximal thoracic aortic redo surgery as a first step for subsequent thoracoabdominal replacement were excluded from this analysis because they represent a different pathology. The institutional review board of the University Hospital of Berne approved the study and waived the need for patient consent.

### Data Collection and Follow-up Protocol

Data were collected prospectively. After surgery, patients were seen in our aortic outpatient clinic on a regular basis. Those who did not show-up in the outpatient consultation, were contacted via general practicioners or directly via phone calls. Consequently, follow-up was complete in all patients.

### Conduction of Extracorporeal Circulation and Myocardial Protection Strategy

According to the anticipated extent of arch involvement and the expected type of repair, patients were cooled to bilateral 20°C tympanic temperature and 26° bladder temperature (more complex repair of the aortic arch) or to bilateral 26°C tympanic temperature and 30° bladder temperature (open distal anastomosis at the proximal level of the arch only). Vasodilators such as nitroprosside and phentolamine are used to achieve homogenous cooling by reducing peripheral vascular resistance. During rewarming target temperatures were bilateral 36°C tympanic temperature as well as 35° core temperature. Cerebral protection was achieved with either selective antegrade perfusion using two perfusion catheters in both common carotid arteries or antegrade perfusion through the right subclavian artery (cannulation site) and an additional catheter in the left common carotid artery. Temperature of the cerebral perfusate was 20°. Total cerebral flow was choosen between 500 and 750 ml/min and targeted according to the anticipated resistance of the cannulas. Most importantly, resistance of 50 mmHg at the level of the individual cannula was not exceeded in order not to expose the brain to episodes of excessive pressure. Myocardial protection was performed using a low-volume cardioplegic solution (Cardioplexol®) as induction cardioplegia with intermittent modified Buckberg cold blood cardioplegia every 20–30 minutes. Before coronary reperfusion, a modified Buckberg warm blood cardioplegia was administered.

After weaning from cardiopulmonary bypass, reversal of heparin with protamine ratio 1∶1 (1 mg protamine per 100 IU heparin) was performed. Intraoperative autologous transfusion using a cell-saver device was used in all patients. Intraoperative and post-operative transfusion thresholds were guided by in-hospital standards supplemented by rotational thrombelastometry (ROTEM, Pentapharm GmbH, Munich, Germany) and analysis of selected parameters of coagulation.

### Definition of Clinical Parameters

Preoperative parameters were defined according to EuroSCORE I and II guidelines [6], [7]. Mortality was defined as in-hospital death. Neurologic injury was defined as any new sensomotoric deficit (including those with subclinical manifestation) persisting at the time of discharge in combination with a morphological correlate in CT-scan or MR imaging (CT). A critical preoperative condition was defined as any one or more of the following: ventricular tachycardia or fibrillation or sudden death with successful resuscitation, preoperative cardiac massage, preoperative ventilation before arrival in the operating theater, preoperative inotropic support, intraaortic balloon counterpulsation or preoperative acute renal failure (anuria or oliguria<10 ml/hour).

### Statistical Methods

Continuous data are presented as the median and interquartile range (range from the 25th to the 75th percentile). Discrete data are given as counts and percentages. Comparisons of continuous data were performed by Mann-Whitney *U* tests, and groups of categorical data were compared by χ^2^ tests.

Overall survival and freedom from reintervention were calculated according to the method of Kaplan and Meier. Univariate regression analysis was performed to assess potential risk factors for in-hospital mortality. A multivariate logistic regression model was then applied to assess the strongest independent risk factor of outcome after adjustment for possible confounding factors. Only variables significant in univariate analysis or imbalanced were considered in the multivariate analysis. Due to the fact that the individual Euro-Score score itself presents a result of a multivariate regression model incorporating more than 10 preoperative variables, the predictive power was assessed independently. Results of the logistic regression model are given as the odds ratio (OR) and the 95% confidence interval (CI) and predictive power was assessed via Receiver-Operating Curve (ROC). Regression diagnostics and overall model fit were performed according to standard procedures. A two-sided *p* value below 0.05 was considered statistically significant. All calculations were performed with SPSS 20.0 software for MacOSX (IBM Inc, Somers, NY).

## Results

### Descriptive Characteristics of the Cohort

Descriptive characteristics of the cohort are shown in **Table 1**. Median age was 60 years (IQR 51–73, 12% female). Thirty-eight percent suffered from coronary artery disease, pulmonary hypertension was present in 12%. Twenty-seven percent had already sustained neurologic injury previously. Median logistic EuroSCORE I levels were 40 (IQR 20–67). Median EuroSCORE II levels were 14 (IQR 10–28).

**Table 1:** Descriptive characteristics of the cohort.

		N overall = 60	
*Demographics*			
	Age, median (IQR)	60	(51–73)
	Female, n (%)	7	(12%)
*Chronic health conditions and risk factors*			
	Hypertension, n (%)	49	(82%)
	Chronic obstructive pulmonary disease, n (%)	7	(12%)
	Diabetes mellitus, n (%)	4	(7%)
	Serum creatinine >200 mmol/l, n (%)	10	(17%)
	Coronary artery disease, n (%)	23	(38%)
	Extracardiac arteriopathy, n (%)	1	(2%)
	Pulmonary hypertension, n (%)	7	(12%)
	Recent myocardial infarction, n (%)	3	(5%)
	Permanent neurologic deficit, n (%)	16	(27%)
	Connective tissue disease, n (%)	4	(7%)
	Logistic EUROSCORE, median (IQR)	40	(20–67)
	Additional EUROSCORE, median (IQR)	12	(10–16)
	EUROSCORE II, median (IQR)	14	(10–28)
*Reason for previous aortic surgery*			
	Aortic dissection, n (%)	33	(55%)
	Isolated aortic aneurysm, n (%)	13	(21%)
	Aortic valve stenosis with ascending disease, n (%)	7	(12%)
	Aortic valve insufficiency with ascending disease, n (%)	4	(7%)
	Other, n (%)	3	(5%)
*Previous surgical approach*			
	Ascending aortic replacement, n (%)	15	(25%)
	Root replacement, n (%)	16	(27%)
	Ascending and (hemi)-arch repair, n (%)	17	(28%)
	Other, n (%)	12	(20%)

Unless otherwise indicated, data are number (percentage). IQR, interquartile range; classification of chronic health conditions and risk factors according to EuroSCORE criteria.

### Reasons for Prior Aortic Surgery and Type of Previous Aortic Operations

Acute aortic dissection was the reason for primary surgery in 55% The remaining primary indications present a combination of aortic valve and root disease with aneurismal involvement of the ascending aorta in various extent and are shown in **Table 1**. Ascending aortic replacement was performed in 25%, root replacement was performed in 27%, 28% received ascending and (hemi)- arch repair. The remaining primary operations are shown in **Table 1**.

### Reasons for Proximal Thoracia Aortic Redo Surgery

The development of an aneurysm following repair of acute aortic dissection was the indication for reoperation in 17% of patients. Aneurysmal progression of primary untreated aortic segments was the indication for reoperation in 13%. A substantial number of patients underwent reoperation due to graft infection or aortic valve endocarditis. Anastomotic aneurysms were the indication for reoperation in 20%. The remaining reasons are shown in **Table 2**. Fifty percent of patients underwent redo surgery on an urgent or emergent basis. Eighty percent of patients underwent primary redo surgery whereas 13.3% underwent their second redo surgery and finally 6.7% underwent their third proximal thoracic aortic redo surgery.

**Table 2:** Surgical characteristics of the cohort.

		N overall = 60	
*Reason for re-intervention*			
	Post- dissection aneurysm, n (%)	10	(17%)
	Aortic aneurysm, n (%)	8	(13%)
	Aortic rupture, n (%)	3	(5%)
	Graft infection/endocarditis, n (%)	21	(35%)
	Anastomotic aneurysms/rupture, n (%)	12	(20%)
	Other, n (%)	6	(10%)
*Timing of re-intervention*			
	Urgent or emergent surgery, n (%)	30	(50%)
*Surgical strategy of reintervention*			
	Axillary/subclavian cannulation, n (%)	24	(40%)
	Direct aortic cannulation, n (%)	27	(45%)
	Partial root/ascending replacement only, n (%)	21	(35%)
	Elephant trunk, n (%)	3	(5%)
	Frozen elephant trunk, n (%)	2	(3%)
	Hemiarch replacement, n (%)	26	(43%)
	Total arch replacement, n (%)	8	(13%)
	Root replacement, n (%)	25	(42%)
	Re-implantation of trunk/arch vessels, n (%)	12	(20%)
*Additional procedures*			
	CABG, n (%)	9	(15%)
	Aortic valve replacement, n (%)	40	(67%)
	Mitral valve repair/replacement, n (%)	6	(10%)
*Duration of re-intervention*			
	ECC in minutes, median (IQR)	150	(85–216)
	Aortic crossclamp times in minutes,median (IQR)	93	(62–137)
	DHCA in minutes, median (IQR)	23	(10–35)

Unless otherwise indicated, data are number (percentage). IQR, interquartile range; classification of chronic health conditions and risk factors according to EuroSCORE criteria.

### Surgical Strategy during Reintervention, Type of Proximal Thoracic Aortic Redo Surgery and Additional Procedures

Sites of cannulation for arterial return during redo surgery are shown in **Table 2**. Forty-two percent of patients underwent aortic root replacement. The distribution of patients undergoing various extent of aortic arch replacement is given in **Table 2**. Additional surgical procedures included aortic valve replacement in 67% (root replacement included), coronary artery bypass grafting (CABG) in 15% and mitral valve repair or replacement in 10%.

### Cardiopulmonary Bypass Data

Median cardiopulmonary bypass times were 150 minutes (IQR 85–216), median aortic cross clamp times were 93 minutes (IQR 52–137) and hypothermic circulatory arrest times with antegrade selective cerebral perfusion were 23 minutes (IQR 10–35).

### Outcome Characteristics of the Cohort

In-hospital mortality was 13%. New onset of neurologic injury was 7%. Acute renal failure requiring intermittent hemodialyis was observed in 10%, pulmonary complications requiring tracheostomy were seen in 3%. The remaining outcome characteristics are shown in **Table 3**.

**Table 3:** Outcome characteristics of the cohort.

		N overall = 60	
*Early in-hospital complications*			
	In-hospital mortality, n (%)	8	(13%)
	Acute renal failure, n (%)	6	(10%)
	Pulmonary complications, n (%)	2	(3%)
	Acute myocardial infarction, n (%)	1	(2%)
	New neurologic deficit, n (%)	4	(7%)
	Sepsis, n (%)	3	(5%)
*Late outcome*			
	Follow-up in months, median (IQR)	23	(1–52)
	Late death, n (%)	13	(22%)
	Need for re-intervention, n (%)	0	(0%)

Unless otherwise indicated, data are number (percentage). IQR, interquartile range; classification of complications according to STS criteria.

### In-hospital Survivors versus Non In-hospital Survivors

Non in-hospital survivors were more likely to have pulmonary arterial hypertension (38% vs. 8%, p = 0.014). These patients were also more likely to present in a critical preoperative state (63% vs. 17%, p = 0.005). Accordingly, additive as well as logistic EuroSCORE **I** levels were higher (17 vs. 12, p = 0.011 and 83 vs. 34, p = 0.004). There was no difference regarding the extent of repair (aortic root replacement 38% vs. 42%, p = 0.80) and the length of hypothermic circulatory arrest (33 minutes vs. 22 minutes, p = 0.14) between surviviors and non-survivors (**Table 4**).

**Table 4:** Distribution of patients by different chronic health conditions and in-hospital risk assessment stratified to in-hospital outcome.

		Death (N = 8) vs. Survival (N = 52)			P
*Demographics*					
	Age, median (IQR)		76 (58–80)	60 (49–70)	0.02
	Female sex, n (%)		2 (25%)	5 (10%)	0.21
*Chronic health conditions and risk factors*					
	Hypertension, n (%)		8 (100%)	41 (79%)	0.17
	Chronic obstructive pulmonary disease, n (%)		2 (25%)	5 (10%)	0.21
	Diabetes mellitus, n (%)		1 (13%)	3 (6%)	0.49
	Serum creatinine >200 mmol/l,n (%)		2 (22%)	8 (15%)	0.50
	Coronary artery disease, n (%)		5 (63%)	18 (35%)	0.14
	Extracardiac arteriopathy, n (%)		0 (0%)	1 (2%)	0.69
	Pulmonary hypertension, n (%)		3 (38%)	4 (8%)	0.014
	Permanent neurologic deficit,n (%)		2 (25%)	14 (27%)	0.90
	Connective tissue disease, n (%)		0 (0%)	4 (8%)	0.44
*Preoperative assessment*					
	Critical preoperative state, n (%)		5 (63%)	9 (17%)	0.005
	Recent myocardial infarction, n (%)		1 (13%)	2 (2%)	0.27
	Logistic EUROSCORE, median, (IQR)		83 (63–95)	34 (20–59)	0.004
	Additive EUROSCORE, median (IQR)		17 (12–23)	12 (10–15)	0.011
	EUROSCORE II, median (IQR)		41 (21–67)	13 (10–20)	0.001
*Surgical management*					
	Root replacement, n (%)		3 (38%)	22 (42%)	0.80
	DHCA in minutes, median (IQR)		33 (24–48)	22 (9–32)	0.14

Unless otherwise indicated, data are number (percentage). IQR, interquartile range; ECC, extracorporal circulation; HCA, hypothermic circulatory arrest; classification of chronic health conditions and risk factors according to EuroSCORE criteria.

### Predictors of Outcome

**Figure 1:**
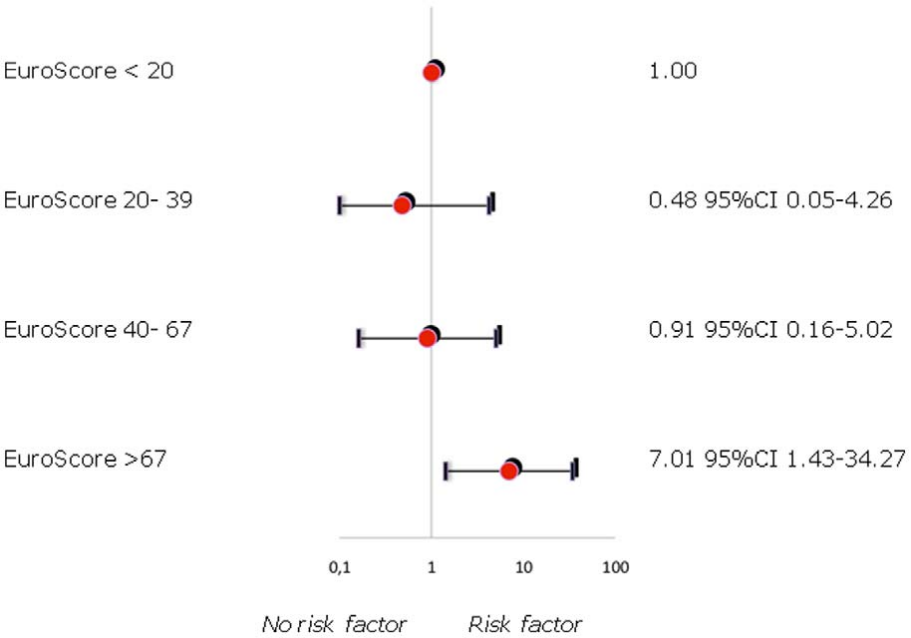
Logistic regression model to assess the predictive power of EuroSCORE I levels.

The preoperative logistic EuroSCORE I confirmed to be a reliable predictor of adverse outcome- (**ROC 0.786**, **95%CI 0.64–0.93**) as did the new EuroSCORE II model: **ROC 0.882 95%CI 0.78–0.98**. Extensive individual logistic EuroSCORE I levels more than 67 showed an **OR** of **7.01**, **95%CI 1.43–34.27** (**Figure 1**). A EuroSCORE II larger than 28 showed an **OR** of **4.44** (**95%CI 1.4–14.06**). Similar results were obtained for the additive EuroSCORE. In individual univariate analysis, the EuroSCORE variable ”critical preoperative state” comprised an **OR** of **7.96 CI 1.61–39.5** but not “advanced age”, as defined older than 65 years of age (**OR 2.67 CI 0.57–12.40**). These findings could be confirmed in multivariable regression analysis (critical preoperative state **OR 7.96 CI 1.51–38.79**; advanced age **OR 2.46 CI 0.48–12.66**; **Hosmer- Lemeshow- test 0.94**).

### Follow-up

**Figure 2:**
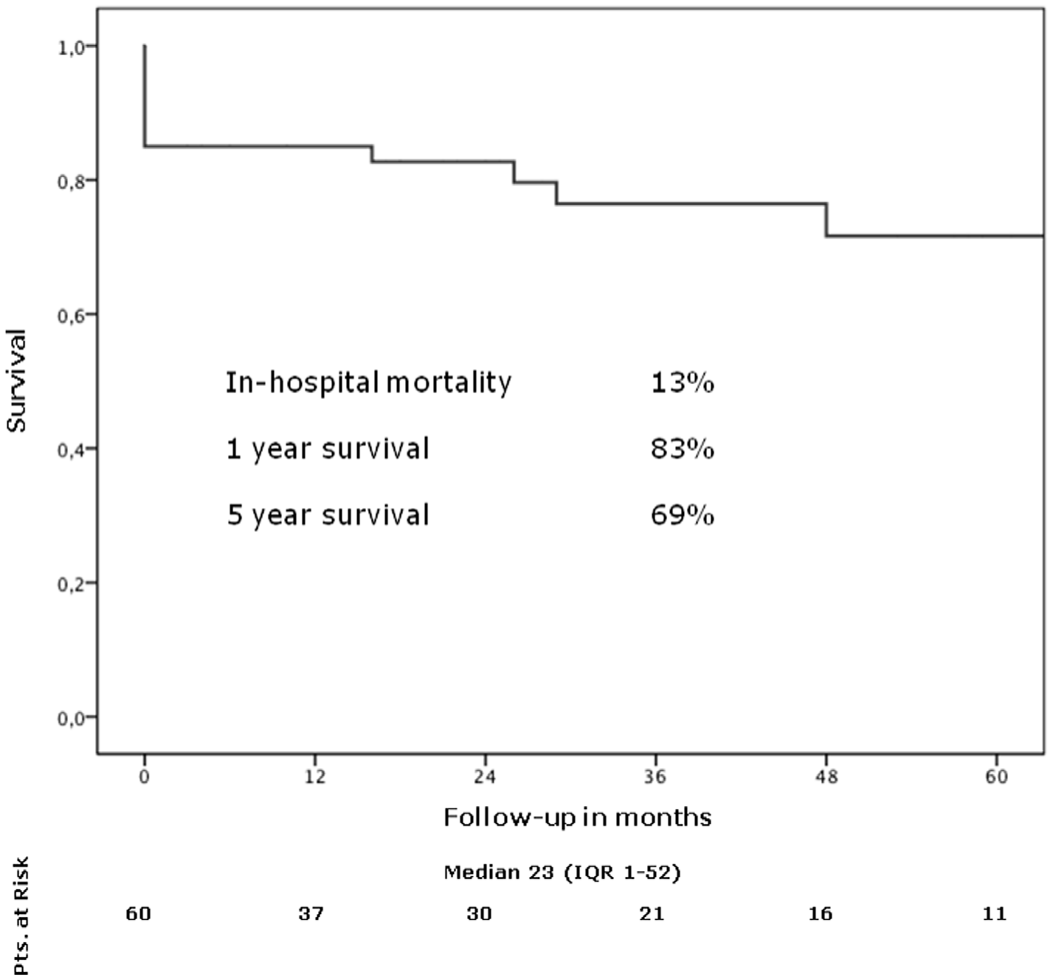
One year and five year actuarial survival rates.

Median follow-up was 23 months (IQR 1–52). One year and five year actuarial survival rates were 83% and 69% respectively (**Figure 2**). Freedom from reoperation during follow-up was 100%.

### Comment

Despite a substantial early attrition rate in patients presenting in a critical preoperative condition, proximal thoracic aortic redo surgery provides excellent early and mid-term results. Higher EuroSCORE I and II levels and a critical preoperative state but not advanced age are independent predictors of in-hospital mortality. As a consequence, age per se should no longer be considered as a contraindication for surgical treatment in this particular group of patients.

The most surprising demographic finding in this cohort was the fact that 27% of patients had already sustained previous neurologic injury. This might well be due to the fact that a substantial percentage of patients had undergone surgery at a time (before 2000) where hypothermic circulatory arrest, especially in the acute setting was still associated with a higher risk of side effects, also due to the fact that selective antegrade cerebral perfusion was not routinely used [8], [9]. In addition, active infective endocarditis (including those with cerebral embolism) was a frequent primary indication for redo-surgery and these two facts might well explain the high percentage of already sustained neurologic injury [9]. The combination of several risk factors also well explains the exceedingly high EuroSCORE levels before proximal thoracic aortic redo sugery. Despite a substiantial number of patients presenting with elevated systolic pulmonary artery pressures, no patients with severe right ventricular impairment were identified accordind to our intraoperative transesophageal echocardiography measurements. However, elevated pulmonary artery pressures have to interpreted as a surrogate of cardiogenic shock in urgent and emergent cases.

The most frequent previous aortic operation was supracoronary repair of acute type A aortic dissection. Evolving knowledge on the natural course of primarily untreated aortic segments (for instance the aortic root) has confirmed that remaining native aortic tissue during primary surgery may well cause secondary dilation, recurrent or new onset of dissection and therefore be the reason for redo surgery within the aortic root [9]. The same may apply to untreated arch segments during primary surgery. However, it remains to be proven that a more aggressive approach at the level of the aortic arch or even the proximal descending thoracic aorta during primary surgery is effective to reduce the need for redo surgery in the future [10]–[12].

Post-dissection aneurysmal formation was the indication in 17% of this patient cohort. From preoperative imaging, limited primary repair with regard to the extent of arch replacement might have been the cause for recurrent pathology. Since open distal anastomosis at the level of the proximal arch is generally accepted as the operation of choice in acute type A aortic dissection, the incidence of secondary arch dilation is expected to diminish in the future. However, a certain percentage of patients will still develop post dissection arch aneurysm despite adequate primary repair. Number and extent of communications between the true and the false channels and evaluation of the functional value of the flow through these communications might help how to decrease the incidence of this problem [13]. Aneurymal degeneration of primary untreated aortic segements was the indication for redo surgery in 13%. The discussion involves the same arguments as those known from post-dissection aneurysm formation. The lessons learned here will probably lead on a more liberal approach to replace the entire aortic root in borderline diameters as well as in patients with bicuspid aortic valves, despite the fact that recommendations in the literature may differ [14]. We are still convinced that the risk benefit ration of complete prophylactic aortic arch replacement does not justify a liberal approach in the arch in patients with regular diameters [15].

Interestingly we have treated a high number of patients with graft infections or with aortic valve endocarditis. This may be due to the fact that patients with this specific problem are referred supra-regional. In these cases we have developed a specific concept of non-alloplastic reconstruction [16]. Aneurysms at the level of a prior anastomosis with and without contained rupture were frequently observed. The majority of those was observed at the sinotubular junction in the non-coronary sinus. As the non-coronary sinus is frequently the weakest one with regard to wall thickness as well as it is the most shear stress-exposed one due hemodynamics and geometry, it is most probably a site of predilection for this problem. A hemi-Yacoub technique in all cases of acute and chronic proximal thoracic aortic pathology in order to replace the non-coronary sinus may help eradicate the problem.

The choice of the most adequate cannulation site might differ from case to case but it is our strategy to have access for arterial return before redo sternotomy and to avoid retrograde arterial perfusion from the iliac region. This strategy may explain our acceptably low rate of perioperative neurologic injury especially in the light of the high rate of priorly sustained preoperative neurologic injury. According to our strategy to excise the maximum of potentially pahological aortic tissue, a high number of root replacements was performed.

In-hospital mortality was substantial but seems acceptable when put in the light of the high risk of the patients treated and therefore performs more than favourable when seen in the light of extremely high EuroSCORE I and II levels. In order to gain better insights into the factors being associated with in-hosptial mortality, we stratified patients into two groups. There was a significant difference with regard to presentation in a critical preoperative state between survivors and non-survivors. This is an important observation as surgeons active in this field do well know the clinical dilemma of decision making in conscious, well-reflected patients asking for treatment or in a situation of a demanding family of an already unconscious patient.

Multivariate logistic regression analysis substantiated the predictive power of higher EuroSCORE I levels with regard to in-hospital mortality. Furthermore, the predictive power of the recently introduced EuroSCORE II higher than 28 could be confirmed in this study. As a consequence, EuroSCORE II may well be used as a valid tool in patients undergoing proximal thoracic aortic redo surgery as a means to objectively predict risk. The same observation was made with regard to the critical preoperative condition. Interestingly and importantly, advanced age was not associated with increased mortality. Several recent papers report that age alone is not a risk factor for major cardiac surgery [10], [17]. This finding might be helpful for physicians faced to elderly patients. Median follow-up in this series was 23 months and the attrition rate during follow-up is well comparable to many other series of patients having been treated for extensive cardiovascular pathology.

### Limitations and Strenghts of the Study

The main limitation of this report is its retrospective, single-center nature. Despite the fact that the cohort for this specific surgical indication is rather large, the sample size is modest as compared to other more frequently observed cardiovascular pathology. However, the results are encouraging in particular, besides the excellent outcome in surviving patients and the full freedom from reoperation during follow-up, with regard to the relatively low number of newly observed neurologic injury in the light of the high number of patients with already sustained neurologic injury before proximal thoracic aortic redo surgery.

In summary, despite a substantial early attrition rate in critically-ill patients, proximal thoracic aortic redo surgery provides excellent early and mid-term results. Higher EuroSCORE I and II levels and a critical preoperative state but not advanced age are independent predictors of in-hospital mortality. As a consequence, age alone should no longer be considered as contraindication for surgery in this particular group of patients.
